# Evaluating Health Information Systems Using Ontologies

**DOI:** 10.2196/medinform.5185

**Published:** 2016-06-16

**Authors:** Shahryar Eivazzadeh, Peter Anderberg, Tobias C Larsson, Samuel A Fricker, Johan Berglund

**Affiliations:** ^1^ Department of Health Science Blekinge Institute of Technology Karlskrona Sweden; ^2^ Department of Mechanical Engineering Blekinge Institute of Technology Karlskrona Sweden; ^3^ Software Engineering Research Laboratory (SERL-Sweden) Blekinge Institute of Technology Karlskrona Sweden; ^4^ Centre for Requirements Engineering (CeRE) i4DS University of Applied Sciences and Arts Northwestern Switzerland Windisch Switzerland

**Keywords:** health information systems, ontologies, evaluation, technology assessment, biomedical

## Abstract

**Background:**

There are several frameworks that attempt to address the challenges of evaluation of health information systems by offering models, methods, and guidelines about what to evaluate, how to evaluate, and how to report the evaluation results. Model-based evaluation frameworks usually suggest universally applicable evaluation aspects but do not consider case-specific aspects. On the other hand, evaluation frameworks that are case specific, by eliciting user requirements, limit their output to the evaluation aspects suggested by the users in the early phases of system development. In addition, these case-specific approaches extract different sets of evaluation aspects from each case, making it challenging to collectively compare, unify, or aggregate the evaluation of a set of heterogeneous health information systems.

**Objectives:**

The aim of this paper is to find a method capable of suggesting evaluation aspects for a set of one or more health information systems—whether similar or heterogeneous—by organizing, unifying, and aggregating the quality attributes extracted from those systems and from an external evaluation framework.

**Methods:**

On the basis of the available literature in semantic networks and ontologies, a method (called Unified eValuation using Ontology; UVON) was developed that can organize, unify, and aggregate the quality attributes of several health information systems into a tree-style ontology structure. The method was extended to integrate its generated ontology with the evaluation aspects suggested by model-based evaluation frameworks. An approach was developed to extract evaluation aspects from the ontology that also considers evaluation case practicalities such as the maximum number of evaluation aspects to be measured or their required degree of specificity. The method was applied and tested in Future Internet Social and Technological Alignment Research (FI-STAR), a project of 7 cloud-based eHealth applications that were developed and deployed across European Union countries.

**Results:**

The relevance of the evaluation aspects created by the UVON method for the FI-STAR project was validated by the corresponding stakeholders of each case. These evaluation aspects were extracted from a UVON-generated ontology structure that reflects both the internally declared required quality attributes in the 7 eHealth applications of the FI-STAR project and the evaluation aspects recommended by the Model for ASsessment of Telemedicine applications (MAST) evaluation framework. The extracted evaluation aspects were used to create questionnaires (for the corresponding patients and health professionals) to evaluate each individual case and the whole of the FI-STAR project.

**Conclusions:**

The UVON method can provide a relevant set of evaluation aspects for a heterogeneous set of health information systems by organizing, unifying, and aggregating the quality attributes through ontological structures. Those quality attributes can be either suggested by evaluation models or elicited from the stakeholders of those systems in the form of system requirements. The method continues to be systematic, context sensitive, and relevant across a heterogeneous set of health information systems.

## Introduction

In one aspect at least, the evaluation of health information systems matches well with their implementation: they both fail very often [1,2,3]. Consequently, in the absence of an evaluation that could deliver insight about the impacts, an implementation cannot gain the necessary accreditation to join the club of successful implementations. Beyond the reports in the literature on the frequent accounts of this kind of failure [3], the reported gaps in the literature [4], and newly emerging papers that introduce new ways of doing health information system evaluation [5], including this paper, can be interpreted as a supporting indicator that the attrition war on the complexity and failure-proneness of health information systems is still ongoing [6]. Doing battle with the complexity and failure-proneness of evaluation are models, methods, and frameworks that try to address what to evaluate, how to evaluate, or how to report the result of an evaluation. In this front, this paper tries to contribute to the answer to *what to evaluate*.

Standing as a cornerstone for evaluation is our interpretation of what things constitute success in health information systems. A body of literature has developed concerning the definition and criteria of a successful health technology, in which the criteria for success go beyond the functionalities of the system [7,8]. Models similar to Technology Acceptance Model (TAM), when applied to health technology context, define this success as the end-users’ acceptance of a health technology system [9]. The success of a system, and hence, the acceptance of a health information system, can be considered the use of that system when using it is voluntary or it can be considered the overall user acceptance when using it is mandatory [10,11].

To map the definition of success of health information systems onto real-world cases, certain evaluation frameworks have emerged [12,6]. These frameworks, with their models, methods, taxonomies, and guidelines, are intended to capture parts of our knowledge about health information systems. This knowledge enables us to evaluate those systems, and it allows for the enlisting and highlighting of the elements of evaluation processes that are more effective, more efficient, or less prone to failure. Evaluation frameworks, specifically in their summative approach, might address what to evaluate, when to evaluate, or how to evaluate [6]. These frameworks might also elaborate on evaluation design, the way to measure the evaluation aspects, or how to compile, interpret, and report the results [13].

Evaluation frameworks offer a wide range of components for designing, implementing, and reporting an evaluation, among which are suggestions or guidelines for finding out the answer to *what to evaluate*. The answer to *what to evaluate* can range from the impact on structural or procedural qualities to more direct outcomes such as the overall impact on patient care [14]. For example, in the STARE-HI statement, which provides guidelines for the components of a final evaluation report of health informatics, the “outcome measures or evaluation criteria” parallel the *what to evaluate* question [13].

To identify evaluation aspects, evaluation frameworks can take two approaches: top down or bottom up. Frameworks that take a top-down approach try to specify the evaluation aspects through instantiating a model in the context of an evaluation case. Frameworks that focus on finding, selecting, and aggregating evaluation aspects through interacting with users, that is, so-called user-centered frameworks, take a bottom-up approach.

In the model-based category, TAM and TAM2 have wide application in different disciplines including health care [7]. Beginning from a unique dimension of *behavioral intention to use (acceptance)*, as a determinant of success or failure, the models go on to expand it to *perceived usefulness* and *perceived ease of use* [15,7], where these two latter dimensions can become the basic constructs of the evaluation aspects. The Unified Theory of Acceptance and Use of Technology (UTAUT) framework introduces 4 other determinants: performance expectancy, effort expectancy, social influence, and facilitating conditions [7]. Of these, the first two can become basic elements for evaluation aspects, but the last two might need more adaptation to be considered as aspects of evaluation for a health information system.

Some model-based frameworks extend further by taking into consideration the relations between the elements in the model. The Fit between Individuals, Task and Technology model includes the *task* element beside the *technology* and *individual* elements. It then goes on to create a triangle of “fitting” relations between these 3 elements. In this triangle, each of the elements or the interaction between each pair of elements is a determinant of success or failure [11]; therefore, each of those 6 can construct an aspect for evaluation. The Human, Organization, and Technology Fit (HOT-fit) model builds upon the DeLone and McLean Information Systems Success Model [16] and extends further by including the *organization* element beside the *technology* and *human* elements [5]. This model also creates a triangle of “fitting” relations between those 3 elements.

Outcome-based evaluation models, such as the Health IT Evaluation Toolkit provided by the Agency for Healthcare Research and Quality, consider very specific evaluation measures for evaluation. For example, in the previously mentioned toolkit, measures are grouped in domains, such as *efficiency*, and there are suggestions or examples for possible measures for each domain, such as *percent of practices or patient units that have gone paperless* [17].

In contrast to model-based approaches, bottom-up approaches are less detailed on about the evaluation aspects landscape; instead, they form this landscape by what they elicit from stakeholders. Requirement engineering, as a practice in system engineering and software engineering disciplines, is expected to capture and document, in a systematic way, user needs for a to-be-produced system [18]. The requirements specified by requirement documents, as a reflection of user needs, determine to a considerable extent what things need to be evaluated at the end of the system deployment and usage phase, in a summative evaluation approach. Some requirement engineering strategies apply generic patterns and models to extract requirements [18], thereby showing some similarity, in this regard, to model-based methods.

The advantages of elicitation-based approaches, such as requirement engineering, result from an ability to directly reflect the case-specific user needs in terms of functionalities and qualities. Elicitation-based approaches enumerate and detail the aspects that need to be evaluated, all from the user perspective. Evaluation aspects that are specified through the requirement engineering process can be dynamically added, removed, or changed due to additional interaction with users or other stakeholders at any time. The adjustments made, such as getting more detailed or more generic, are the result of new findings and insights, new priorities, or the limitations that arise in the implementation of the evaluation.

The advantages in the requirement engineering approach come at a cost of certain limitations compared with model-based methods. Most of the requirement elicitation activities are accomplished in the early stages of system development, when the users do not have a clear image of what they want or do not want in the final system [19]. However, a model-based approach goes beyond the requirements expressed by the users of a specific case by presenting models that are summaries of past experiences in a wide range of similar cases and studies.

Being case-specific by using requirement engineering processes has a side effect: the different sets of evaluation aspects elicited from each case, which can even be mutually heterogeneous. Model-based approaches might perform more uniformly in this regard, as they try to enumerate and unify the possible evaluation aspects through their models imposing a kind of unification from the beginning. However, there still exists a group of studies asking for measures to reduce the heterogeneity of evaluation aspects in these approaches [12].

Heterogeneity makes evaluation of multiple cases or aggregation of individual evaluations a challenge. In a normative evaluation, comparability is the cornerstone of evaluation [20]), in the sense that things are supposed to be better or worse than one another or than a common benchmark, standard, norm, average, or mode, in some specific aspects. Without comparability, the evaluation subjects can, at best, only be compared with themselves in the course of their different stages of life (longitudinal study).

In health technology, the challenge of heterogeneity for comparing and evaluation can be more intense. The health technology assessment literature applies a very inclusive definition of *health technology*, which results in a heterogeneous evaluation landscape. The heterogeneity of evaluation aspects is not limited to the heterogeneity of actors and their responses in a health setting; rather, it also includes the heterogeneity of health information technology itself. For example, the glossary of health technology assessment by the International Network of Agencies for Health Technology Assessment (INAHTA) describes health technology as the “pharmaceuticals, devices, procedures, and organizational systems used in health care” [21]. This description conveys how intervention is packaged in chemicals, supported by devices, organized as procedures running over time, or structured or supported by structures in organizational systems. Similarly, inclusive and comprehensive definitions can be found in other studies [22,23]. This heterogeneous evaluation context can create problems for any evaluation framework that tries to stretch to accommodate a diverse set of health technology implementations. This heterogeneity can present challenges for an evaluation framework in comparing evaluation aspects [24] and, consequently, in summing up reports [25] as well as in the creation of unified evaluation guidelines, and even in the evaluation of the evaluation process.

By extracting the lowest common denominators from among evaluation subjects, thereby creating a uniform context for comparison and evaluation, we can tackle the challenge of heterogeneity via elicitation-based evaluation approaches. Vice versa, the evaluation aspects in an evaluation framework suggest the common denominators between different elements. The lowest common denominator, as its mathematical concept suggests, expands to include elements from all parties, where the expansion has been kept to the lowest possible degree.

Usually, there are tradeoffs and challenges around the universality of an evaluation aspect related to how common it is and its relativeness (ie, how low and close to the original elements it lies). When the scopes differ, their nonoverlapped areas might be considerable, making it a challenge to find the common evaluation aspects. Furthermore, the same concepts might be perceived or presented differently by different stakeholders [26]. In addition, different approaches usually target different aspects to be evaluated, as a matter of focus or preference.

It is possible to merge the results of model-centered and elicitation-centered approaches. The merged output provides the advantages of both approaches while allowing the approaches to mutually cover for some of their challenges and shortcomings.

The aim of this paper is to address the question of *what to evaluate* in a health information system by proposing a method (called Unified eValuation using Ontology; UVON) which constructs evaluation aspects by organizing quality attributes in ontological structures. The method deals with the challenges of model-based evaluation frameworks by eliciting case-specific evaluation aspects, adapting and integrating evaluation aspects from some model-based evaluation frameworks and accommodating new cases that show up over time. The method can address heterogeneity by unifying different quality attributes that are extracted from one or more evaluation cases. This unification is possible with some arbitrary degree of balance between similarities and differences with respect to the needs of evaluation implementation. As a proof of the applicability of the proposed method, it has been instantiated and used in a real-world case for evaluating health information systems.

The structure of the rest of this paper is as follows. The research method that resulted in the UVON method is described in Methods section. The result, that is, the UVON method, is covered in The UVON Method for Unifying the Evaluation Aspects section, whereas its application in the context project is covered in Result of the UVON Method Application in the FI-STAR Project section. The rationale behind the method is discussed in Discussion section and the possible extensions and limitations are found in Extending the Evaluation Using the Ontology and Limitations of the UVON Method sections. The Conclusions section summarizes the conclusions of the paper.

## Methods

### The FI-STAR case

The FI-STAR project is a pilot project in eHealth systems funded by the European Union (EU). The evaluation of the FI-STAR project has been the major motive, the empirical basis, and the test bed for our proposed evaluation method, that is, the UVON method (to be described in Results section). FI-STAR is a project within the Future Internet Public-Private Partnership Programme (FI-PPP) and relates to the Future Internet (FI) series of technology platforms. The project consists of 7 different eHealth cloud-based applications being developed and deployed in 7 pilots across Europe. Each of these applications serves a different community of patients and health professionals [27] and has different expected clinical outcomes. FI-STAR and its 7 pilot projects rose to the challenge of finding an evaluation mechanism that can be used both to evaluate each project and to aggregate the result of those evaluations as an evaluation of the whole FI-STAR project.

### Research Method

A general review of the existing evaluation frameworks was done. Existing model-based evaluation frameworks, which usually suggest universal quality attributes for evaluation, could not cover all the quality attributes (ie, evaluation aspects) reflected by the requirement documents of the pilot projects in FI-STAR. Even if there was a good coverage of the demanded evaluation aspects, there was still no guarantee that they could maintain the same degree of good coverage for the future expansions of the FI-STAR project. On the other hand, the requirement documents from the FI-STAR project were not expected to be the ultimate sources for identifying those quality attributes. It was speculated that there could exist other relevant quality attributes that were captured in the related literature or embedded in other, mostly model-based, health information system evaluation frameworks. For these reasons, it was decided to combine quality attributes both from the FI-STAR sources and a relevant external evaluation framework. To find other relevant evaluation aspects, a more specific review of the current literature was performed that was more focused on finding an evaluation framework of health information systems that sufficiently matched the specifications of the FI-STAR project. The review considered the MAST framework [28] as a candidate evaluation framework. This evaluation framework was expected to cover the quality attributes that were not indicated in the FI-STAR requirement documents but that were considered necessary to evaluate in similar projects. These extra quality attributes are suggested by expert opinions and background studies [28]. Nevertheless, it was necessary to integrate the quality attributes extracted from this framework with the quality attributes extracted from the FI-STAR requirement documents.

Regarding the heterogeneity of FI-STAR’s 7 pilot projects, an evaluation mechanism was needed to extract common qualities from different requirement declarations and unify them. A review of the related literature showed that the literature on ontologies refers to the same functionalities, that is, capturing the concepts (quality attributes in our case) and their relations in a domain [29]. It was considered that subclass and superclass relations and the way they are represented in ontology unify the heterogeneous quality attributes that exist in our evaluation case. For the purposes of the possible future expansions of the FI-STAR project, this utilization of ontological structures needed to be systematic and easily repeatable.

## Results

A method was developed to organize and unify the captured quality attributes via requirement engineering into a tree-style ontology structure and to integrate that structure with the recommended evaluation aspects from another evaluation framework. The method was applied for the 7 pilots of the FI-STAR project, which resulted in a tree-style ontology of the quality attributes mentioned in the project requirement documents and the MAST evaluation framework. The top 10 nodes of the tree-style ontology were chosen as the 10 aspects of evaluation relevant to the FI-STAR project and its pilot cases.

### The UVON Method for Unifying the Evaluation Aspects

Methodical capture of a local ontology [30] from the quality attributes, that is, evaluation aspect ontology and reaching unification by the nature of its tree structure is the primary strategy behind our method. Therefore, the UVON method is introduced, so named to underline *Unified eValuation* of aspects as the target and *ONtology* construction or integration as the core algorithm. The ontology construction method presented in this paper is a simple, semiautomated method, configured and tested against FI-STAR project use cases. The UVON method does not try to introduce a new way of ontology construction; rather, it focuses on how to form a local ontology [30,31] out of the quality attributes of a system and use it for the purpose of finding out what to evaluate. In this regard, the ontology construction in the UVON method is a reorganization of common practices, such as those introduced by [29].

The ontology structure, in its tree form, is the backbone of the UVON method. Modern ontology definition languages can show different types of relations, but for the sake of our method here, we only use the *is of type* relation. The *is of type* relation can also describe pairs such as parent and child, superclass and subclass, or general and specific relations. This kind of relation creates a direct acyclic graph structure, which is or can be converted to a tree form. In this tree, the terms and concepts are nodes of the tree. The branches consist of those nodes connected by *is of type* relations. The tree has a root, which is the superclass, parent, or the general form of all other nodes. Traditionally, this node has been called the *thing* [29].

**Figure 1:**
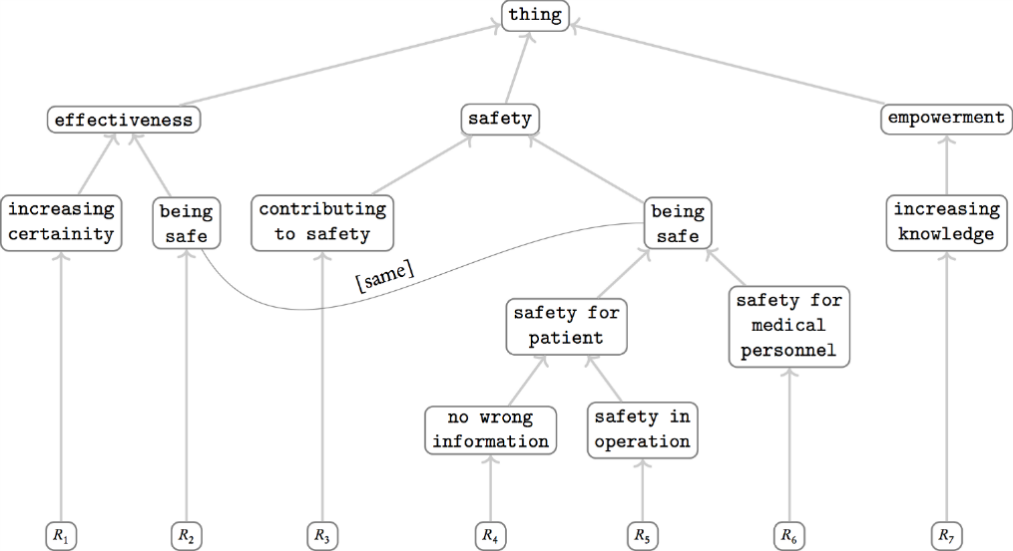
An example snapshot of the output ontology while running the UVON method.

Figure 1 is an example of how this ontology structure can look. All the nodes in this picture are quality attributes, except the leaf nodes at the bottom, which are instances of health information systems. While going up to the top layers in the ontology, the quality attributes become more generic, at the same time aggregating and unifying their child nodes.

**Figure 2:**
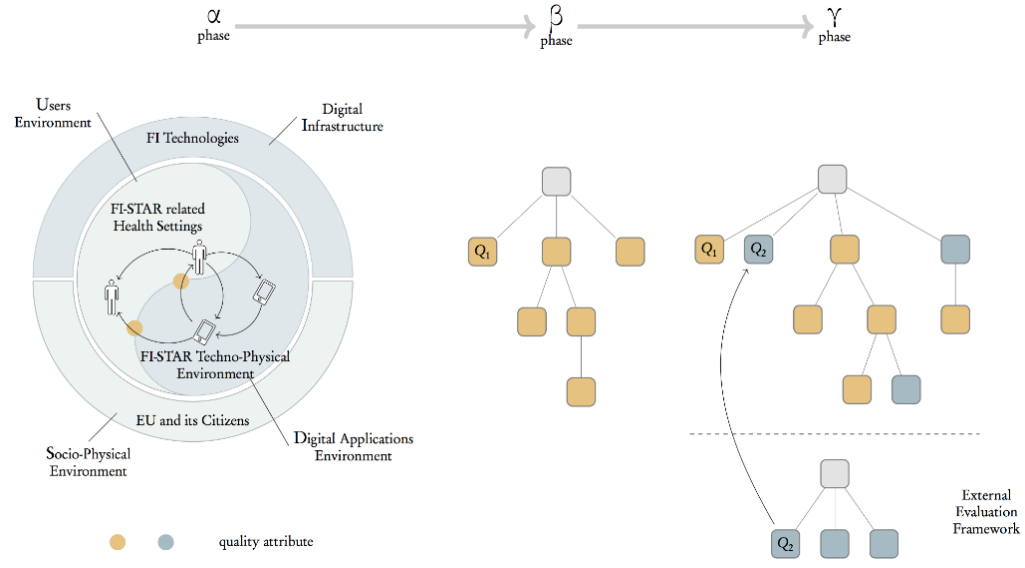
Ontology construction for a health information system.

The UVON method is composed of 3 phases: α, β, and γ (Figure 2). In the first phase, all quality attributes elicited by the requirement engineering process are collected in an unstructured set that is respectively called α set. In the next phase (β), based on the α set, an ontology is developed by the UVON method, which is called β (beta) ontology. In the next step, if the ontology is extended by an external evaluation framework (as discussed in the method), then it is called γ (gamma) ontology.

The β ontology construction begins with a special initial node (ie, quality attribute) that is called *thing*. All the collected quality attributes are going to begin a journey to find their position in the ontology structure, beginning from the *thing* node and going down the ontology structure to certain points specified by the algorithm. This journey is actually a depth-first tree traversal algorithm [32] with some modifications. To avoid confusion in the course of this algorithm, a quality attribute that seeks to find its position is called a *traveling quality attributes* or Q_t.

The first quality attribute simply needs to add itself as the child of the *thing* root node. For the remaining quality attributes, each checks to see if there exists any child of the *thing* node, where the child is a superclass (superset, super concept, general concept, more abstract form, etc) with regard to the traveling quality attribute (Q_t). If such a child node (quality attribute) exists (let’s say Q_n) then the journey continues by taking the route through that child node. The algorithm examines the children of Q_n (if any exist) to see if it is a subclass to any of them (or they are superclass to Q_t).

The journey ends at some point because of the following situations: If there is no child for a new root quality attribute (Q_n), then the traveling quality attribute (Q_t) should be added as a child to this one and its journey ends. That is the same if there exist children to a new root quality attribute (Q_n), but any of them is neither a superclass nor a subclass to our traveling quality attribute. Beside these two situations, it is possible that no child is a superclass, but one or more of them are the subclass of the traveling quality attribute (Q_t). In this situation, the traveling quality attribute (Q_t) itself becomes a child of that new root quality attribute, and those child quality attributes move down to become children of the traveling quality attribute (Q_t).

To keep the ontology as a tree, if a traveling quality attribute (Q_t) finds more than one superclass child of itself in a given situation, then it should replicate (fork) itself into instances, as many as the number of those children, and go through each branch separately. It is important to note that, logically, this replication cannot happen over two disjoint (mutually exclusive) branches. It is also possible to inject new quality attributes in between a parent node and children, but only if it does not break subclass or superclass relations. This injection can help to create ontologies in which the nodes at each level of the tree have a similar degree of generality, and each branch of the tree grows from generic nodes to more specific ones.

This customized depth-first tree traversal algorithm, which actually constructs a tree-style ontology instead of just traversing one, is considered semiautomated, as it relies on human decision in two cases. The first case is when it is needed to consider the superclass to subclass relations between two quality attributes. The gradual development of the ontology through the UVON method spreads the decision about superclass to subclass relations across the course of ontology construction. The unification of heterogeneous quality attributes (nodes) is the result of accumulating these distributed decisions, which are embodied as superclass to subclass relations. Each of these relations (ie, decisions) makes at least 2 separate quality attributes closer together by representing them through more generic quality attributes.

In addition, one can inject a new quality attribute to the ontology tree, although that quality attribute is not explicitly mentioned in the requirement documents. This injection is only allowed when that quality attribute summarizes or equals a single or a few sibling quality attributes that are already in the ontology. The injection can improve clarity of the ontology. It can also help adjust the branches of the ontology tree to grow to a certain height, which can be helpful when a specific level of the tree is going to be considered as the base for creating a questionnaire. This adjustment of branch height might be needed if a branch is not tall enough to reach a specific level, meaning none of the quality attributes in that branch gets presented in the questionnaire. In addition, if a quality attribute is very specific compared with other quality attributes in that level of the tree, the questions in the questionnaire become inconsistent in their degree of generality. This inconsistency can be handled by injecting more generic quality attributes above the existing leaf node in the branch. All the previously mentioned benefits come with the cost of subjectivity in introducing a new quality attribute.

The γ phase ontology is constructed the same as the β phase, but it adds materials (quality attributes) from external sources. In this sense, the quality attributes specified in an external evaluation framework, probably a model-based one, should be extracted first. Those quality attributes should be fed into the β ontology the same as other quality attributes during the β phase. The UVON method does not discriminate between quality attribute by the origin, but it might be a good practice to mark those quality attributes originally from the external evaluation framework if we need later to make sure they are used by their original names in the summarizing level (to be discussed in the following paragraphs).

Each level of the resulting ontology tree(s)—except those that are deeper than the length of the shortest branch—represents or summarizes quality attributes of the whole system in some degree of generality or specificity. That of the *root* node is the most general quality attribute, which is too general to be useful for any evaluation; as for the levels below, each gives a view of the quality attributes in the whole system. As each parent node represents a general form of its children, each level summarizes the level below. We refer to one of these levels of the ontology tree that is considered for creating a questionnaire as the *summarizing level*.

**Figure 3:**
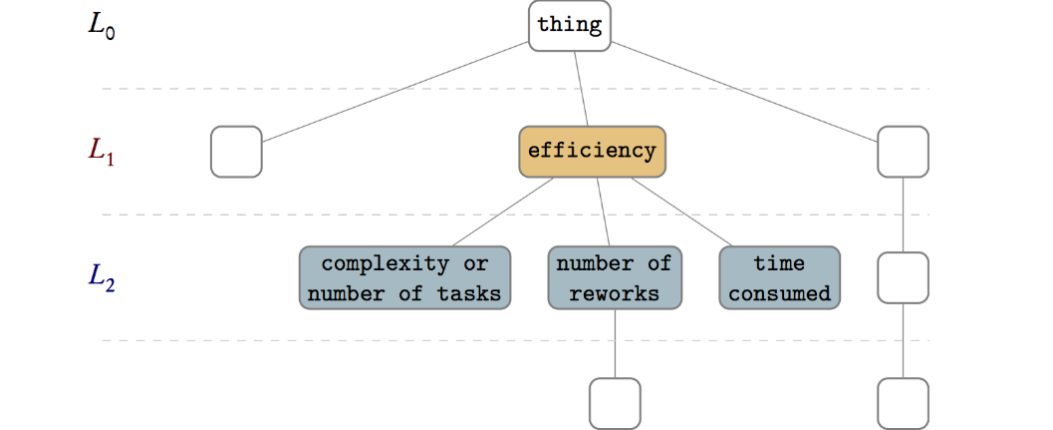
More details can be evaluated by looking at deeper nodes in the ontology structure.

The quality attributes in each of the other levels (such as L_1 in Figure 3) can be evaluation aspects (ie, the answer to *what to evaluate*) that can be measured by a questionnaire or other measurement methods. In addition, depending on the measuring method, the level below the summarizing level can be used to give details for each of the evaluation aspects. The practicalities of measurement in a case determine which summarizing level to choose. Levels closer to the root can be too abstract, whereas deeper levels can be too detailed. In addition, the number of quality attributes in a level can impact which level is appropriate. In the FI-STAR project, the limitation on the number of questions in the questionnaire was a determinant for selecting the summarizing level, where only level 2 fit the project limitations (although level 3 helped to make each question more detailed). It is possible to grow a short branch by adding a chain of children that are the same as their parents to make the branch reach a specific level, thereby making that level selectable as a summarizing level.

### Result of the UVON Method Application in the FI-STAR Project

Harvesting the value-cases and requirement documents for all 7 trial-cases in the FI-STAR project provided the initial set of quality attributes, that is, the α set. Several quality attributes were redundant or similar, but it was left to the UVON method to unify them. There were also several quality attributes with the same wording but different conceptual indications in their respective usage contexts. These quality attributes we added to the α set with small modifications to differentiate them from each other. For example, 2 different references to *efficiency* were converted to *efficiency by reducing complexity* and *efficiency by reducing time*.

In the next step, that is, β phase, the UVON method developed β ontology by using the α set. The redundant quality attributes were integrated into single entities, whereas other quality attributes were grouped by their direct or indirect parents in the ontology structure regarding their degree of similarity or dissimilarity.

In addition, it was noticed that quality attributes are preferred—although not necessarily always—to be noun phrases rather than adjective phrases; this is because fulfilling a quality attribute expressed in an adjective phrase could imply that all of its child quality attributes need to be fulfilled. For example, to fulfill the quality of being *safe*, it is required to be both *safe for patient* and *safe for medical personnel*. This is in contrast to the *child is type of parent* relations that exist between the ontology entities. However, if we consider the noun form (noun phrase), that is, *safety* rather than *safe*, then *safety for patient* and *safety for medical personnel* are all subtopics of *safety*; hence, that would be correct and more intuitive. In addition, considering that each node in the ontology is an aspect for evaluation can make deciding parent-child relations more straightforward. For example, the *safety* node should be read as *safety aspect*, and its child should be read as *safety for patient aspect*.

Applying the UVON method in its β and γ phases, respectively, created the β and γ ontology structures (γ in Multimedia Appendix 1). The first ontology structure (β) is based on the α set of collected quality attributes, whereas the second one (γ) extends the β ontology by integrating the MAST framework evaluation aspects (grouped as domains) as specified by MAST [28]. Here, “integration is the process of building an ontology in one subject reusing one or more ontologies in different subjects” [33]. In this sense, γ ontology is constructed by mapping, aligning, or merging [34] the ontological representation of the external framework evaluation aspects (MAST in our case) to the β ontology. The result of the integration is shown in Table 1.

The MAST framework specifies 7 evaluation domains, where each contains several topics (aspects or sub-aspects) [28]. Due to the FI-STAR project requirements, we ignored *clinical effectiveness* and *sociocultural*, *ethical, and legal* domains (These were the job of other teams). One other domain, *health problem and description of the application* and some aspects in other domains could not be considered as quality attributes and were removed from the process. The remaining 4 domains that were fed into the UVON method are safety, patient perspectives, economic aspects, and organizational aspects. There was an interesting observation, a possible motivation for further investigations: the aspects in those 4 domains overlap considerably with the evaluation aspects that were elicited from FI-STAR users and formed into an ontology by the UVON method.

Both the β and γ ontology structures were described in Web Ontology Language (OWL) using Protégé version 4.x software. OWL, as an ontology language, can describe a domain of knowledge through its lingual elements and their relations [35]. In OWL, there exist individuals, classes, class relations, individual relations, and relation hierarchies [36]. In FI-STAR ontology structures, the individuals were mapped to the use-cases in the FI-STAR project; classes were used to represent quality attributes (i.e., the evaluation aspects); and class relations became the hierarchal relations between quality attributes (ie, *is of type* or the superclass to subclass relations). Individual relations and relation hierarchies were not used.

**Table 1:** The mapping between MAST evaluation aspects and the final evaluation aspects for the FI-STAR project using UVON.

MAST		Final top aspect
Domains	Aspects	
**Health problem and description of the application**		^a^
**Safety**		
	Clinical safety (patients and staff)	Safety
	Technical safety (technical reliability)	Safety
**Clinical effectiveness**		^b^
	Effects on mortality	^b^
	Effects on morbidity	^b^
	Effects on health-related quality of life (HRQL)	^b^
	Behavioral outcomes	^b^(but can relate to adhereability)
	Usage of health services	^b^(but can relate to adhereability)
**Patient perspectives**		
	Satisfaction and acceptance	^c^
	Understanding of information	Accessibility
	Confidence in the treatment	Trustability and authenticity
	Ability to use the application	Accessibility
	Access and accessibility	Accessibility
	Empowerment, self-efficacy	Empowerment
**Economic aspects**		
	Amount of resources used when delivering the application and comparators	Efficiency
	Prices for each resource	Efficiency
	Related changes in use of health care	^a^
	Clinical effectiveness	^b^
	Expenditures per year	Affordability
	Revenue per year	^b^
**Organizational aspects**		
	Process	^a^(but can relate to efficiency)
	Structure	^a^
	Culture	^a^
**Sociocultural, ethical, and legal aspects**		^b^

^a^Not a quality attribute.

^b^Not included because of the FI-STAR project definition and division of tasks.

^c^Had been already covered by some generic questions in the output questionnaire.

Some generic nodes were inserted to group sibling nodes that were conceptually closer together in the ontology structure. If a quality attribute was connected to 2 different branches, it was forked and presented in the both branches (as described before); that keeps the ontology in a tree structure rather than an acyclic directed graph.

Applying the UVON method in the FI-STAR project case, at the end of the γ phase, 10 nodes appeared below the root of the ontology tree (Textbox 1). These 10 quality attributes at the second level of the tree are parents to other child nodes; therefore, each is the unification and aggregation of other quality attributes that were originated either in the FI-STAR requirement documents or the MAST framework and reside below these 10 quality attributes. The number 10 was within the scope of practical considerations for creating an evaluation questionnaire for the FI-STAR project, but we also considered the third level of the tree to provide more details for each question in the questionnaire. Due to separation of responsibilities in the FI-STAR project, these 10 quality attributes do not represent other aspects such as the *clinical effectiveness* or *legal and ethical* ones. The number could have been larger than 10 if we had included those aspects when applying the UVON method in the project.

**The list of quality attributes appearing in the second level of the ontology using the UVON method in the FI-STAR project.**

Quality name

- Accessibility
- Adhereability
- Affordability
- Authenticity
- Availability
- Efficiency
- Effectiveness
- Empowerment
- Safety
- Trustability

In the FI-STAR project, the measurement of evaluation aspects was performed through a questionnaire based on those 10 extracted aspects in the γ ontology. Two versions of the questionnaire had been created: one for the patients and one for the health professionals, where each expressed the same concept in 2 different wordings (Note: one operation theatre case did not have patient questionnaire).

**Figure 4:**
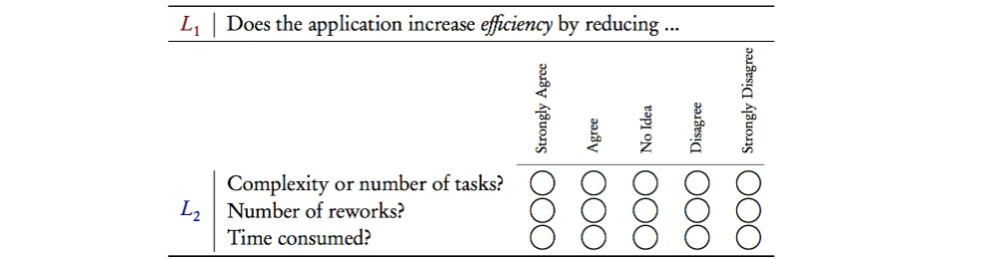
Sample questionnaire output from the UVON method.

Generally and regarding practicalities of an evaluation case, it is possible to consider deeper levels of the resulting γ ontology in a given case. In the FI-STAR case, this possibility is reflected in a sample question on *efficiency* from the questionnaire (Figure 4), where a general question got more detailed by considering other quality attributes below the second level of the ontology. This possibility of going deeper is also depicted in Figure 3.

In the FI-STAR project, the quality attributes (and later the questionnaires) were delivered to each case’s stakeholders, who were asked to validate the relevancy of each quality attribute or the corresponding question regarding their case. All the cases in the FI-STAR project validated and approved their relevancy, whereas some asked for minor changes in the wordings of some of the questions to be clearer for the patient respondents in their case.

## Discussion

Ontologies are formal and computable ways of capturing knowledge in a domain—whether local or global [30]—by specifying the domain’s key concepts (or objects) and interconnecting them by a predefined set of relations [29]. Formality and computability help to communicate knowledge between people or software agents, enable reuse of knowledge, make explicit declaration of the assumptions, and facilitate the analysis and study of the domain knowledge [29]. Inference algorithms can infer and extract new knowledge or predict or deduce new situations by analyzing an ontology. As reflected in the previously mentioned ontology description, an ontology is structured as a network (mathematically a graph). Limiting the kind of relations between the concepts might result in specific structural forms such as trees.

An ontology would be formed as a hierarchy if the relations between the concepts are limited to the *is of type* relation, where each nonleaf concept is a more generic form or superclass to its children. This hierarchy can be an *acyclic direct graph* if we allow one concept to be a subclass of more than one other concept, and it would be a *tree* if one concept is a subclass of only one other concept. The acyclic directed graph can be converted to a tree if we replicate the same concept-leaf in different branches. The unification that exists in the nature of a tree graph, that is, unification of branches toward the root, is the source of unification that we want to apply for the evaluation of quality attributes in health information systems; that is why the UVON method creates this type of structure.

Ontologies are traditionally the output of manual content curation and its associated consensus-establishment processes [37]. Nevertheless, automated or semiautomated methods of ontology construction might reveal considerable advantages in efficiency, repeatability, and uniformity. The UVON method described in this paper uses a semiautomatic approach toward creating tree-style ontologies for the sake of extracting evaluation aspects.

### Extending the Evaluation Using the Ontology

The ontological representation of a health information system gives a computable structure from which several indications, including evaluation aspects, can be extracted. Functions can be defined on this ontology that quantify, combine, compare, or select some of the nodes or branches. The ontology itself can be extended by assigning values to its nodes and edges, giving the possibility of further inferences. For example, if 2 nodes (quality attributes) are disjoint (mutually exclusive), any 2 children from each of them would be disjoint, respectively. If during the application of the UVON method, by mistake, one quality attribute were replicated into 2 disjoint branches, then this mistake can be detected and avoided automatically (replication would be disallowed between those specific nodes).

As discussed in “Result of the UVON Method Application in the FI-STAR Project” section and shown in Table 1, we skipped the *clinical effectiveness* and *sociocultural, ethical, and legal* domains from the MAST framework due to the project definition. Nevertheless, the UVON method can consider those aspects when they are applicable and there are no project restrictions. Therefore, we hope to witness more inclusive applications of the UVON method in the future cases.

In addition, the selection of the MAST framework was due to its common themes with the eHealth applications in the FI-STAR project. We encourage application of the UVON method by considering other relevant evaluation frameworks, not necessarily MAST. The results of those applications can demonstrate the powers, weaknesses, and extension points of the FI-STAR method.

The UVON method is context-insensitive in its approach. Still, more empirical evidence, with a higher degree of diversity, is needed to examine what the challenges or advantages of applying the UVON method are in a more diverse range of fields beyond health information systems.

### Limitations of the UVON Method

The UVON method is subject to conceptual and methodological limitations in its capacities. Probably, a prominent conceptual limitation is the fact that the method does not represent or give an account of the dynamics of the health information systems; hence, it cannot facilitate their evaluation. The relations in the UVON-constructed ontologies are restricted to the *is of type* relationship and cannot reflect how qualities or other indicators impact each other. The absence of insight about the dynamics of a health information system prevents predictive evaluations. In consequence, any emergent behavior that is not explicitly captured by requirement documents or the to-be-merged external evaluation framework is going to be ignored. From the other side, it can still be imagined that the output ontologies of the UVON method can be used as scaffolds in models that incorporate dynamics of health information systems.

The UVON method partially relies on subjective decision-making, which can create methodological limitations and challenges. Although the main strategy in the UVON method is to minimize these subjective decisions, the existing ones can still result in creating different ontologies in different applications of the method. As a suggestion, for the sake of reaching more convergence, it is possible to think of enhancing the method with more objective lexical analytical methods. Methods of ontology construction and integration, especially those concerning class inheritance analysis [34], can be valid candidates for these types of methods.

UVON-generated ontologies are not advised for universal application. However, for a new case of evaluation, a UVON-generated ontology that was developed for similar cases can be considered as an alternative to developing a new ontology with consideration to project resource limitations. This reuse should be accomplished with due consideration to the fact that quality attributes of the same wording might indicate slightly different meanings in different cases. This case-sensitivity of meanings might result in different subclass and superclass relations, changing the structure of the ontology and making the reuse of the unadjusted ontology problematic.

The UVON method cannot guarantee that in the output ontology each of the branches that begin from the root will reach the level of the tree (that is, have a node at that level) where we want to base our questionnaire (or any other measurement method). Hence, a short branch might need to be extended to appear at some specific tree level where the questionnaire is based. In addition, the method does not guarantee that the quality attributes in that level are all of the same degree of generality of specificity. It is also not guaranteed that the number of nodes (quality attributes) at any level matches the practicalities of evaluation; there can be too few or too many. For example, in the FI-STAR case, the number of quality attributes in the target level (level 2) had to match with the appropriate maximum number of questions that could be put in a questionnaire; fortunately, it was within the boundaries.

It is also possible, at least in theory, that all quality attributes end up being a direct child of the root *thing* node. The resultant dwarf and horizontally inflated ontology structure does not unify any of the child quality attributes; hence, the method output would be useless. The methodological limitations can result in the need for manual adjustments, such as adding extra nodes between some parent-child nodes. Of course, the manual adjustments can add more subjectivity into the formation of the ontologies.

The UVON method permits integrating evaluation aspects from other evaluation frameworks. Still, it does not guarantee that the result will include all features of the integrated evaluation framework. Still, this integration involves the suggested evaluation aspects of those evaluation frameworks. If a framework dynamically changes its suggested evaluation aspects, for example, based on the evaluation case specifications, the UVON does not follow that dynamic feature. In addition, the straightforward wordings for an evaluation aspect in an evaluation framework might be obscured by going through the integration process in the UVON method, being replaced by more generic terms.

### Conclusion

The unifying nature of ontologies, when they are in tree form, can be used to create a common ground of evaluation for heterogeneous health technologies. Ontologies can be originated from requirement and value-case documents, that is, internal; they can be extracted from available external evaluation frameworks, that is, external; or they can be originated from a mix of both internal and external sources. The UVON method introduced in this paper was able to create a common ground for evaluation by creating an ontology from requirement and value-case documents of the 7 trial projects in the FI-STAR project and extend that ontology by mixing elements from the MAST evaluation framework. The UVON method can be used in other, similar cases to create ontologies for evaluation and to mix them with elements from other evaluation frameworks.

The UVON method stands in contrast with other methods that do not consider case-specific internal requirements or cannot be easily extended to include other evaluation frameworks. The ontological structure of evaluation aspects created by the UVON method offers the possibility of further investigations for other indications related to evaluation of the subject systems.

The final result of applying the UVON method in the FI-STAR project resulted in 10 evaluation aspects to be chosen for measurement. This set of evaluation aspects can grow adaptively to project changes, be repeated in similar cases, and be a starting point for future evaluations in similar projects. By applying the UVON method in more cases, a possible stable result can be suggested for the set of generic evaluation aspects that are usable in evaluation cases similar to FI-STAR.

## Appendix

Multimedia Appendix 1 UVON-generated Ontology (in OWL) for the FI-STAR project.

## Abbreviations

EU: European Union
FI: Future Internet
FI-STAR: Future Internet Social and Technological Alignment Research
FI-PPP: Future Internet Public-Private Partnership Programme
FITT: Fit between Individuals, Task and Technology
HOT-fit: Human, Organization, and Technology Fit
INAHTA: International Network of Agencies for Health Technology Assessment
MAST: Model for Assessment of Telemedicine applications
OWL: Web Ontology Language
STARE-HI: Statement on the Reporting of Evaluation studies in Health Informatics
TAM: Technology Acceptance Model
TAM2: Technology Acceptance Model 2
UTAUT: Unified Theory of Acceptance and Use of Technology
UVON: Unified eValuation using Ontology
